# *Bacillus velezensis* 20507 promotes symbiosis between *Bradyrhizobium japonicum* USDA110 and soybean by secreting flavonoids

**DOI:** 10.3389/fmicb.2025.1572568

**Published:** 2025-03-27

**Authors:** Yunqing Cheng, Xingtong Jiang, Xinyi He, Zhaoyang Wu, Qiang Lv, Shuang Zhao, Xinyue Zhang, Shuai Wang, Hongli He, Jianfeng Liu

**Affiliations:** Jilin Provincial Key Laboratory of Plant Resource Science and Green Production, Jilin Normal University, Siping, China

**Keywords:** soybean, transcriptome, growth, nitrogen fixation, nodulation

## Abstract

**Introduction:**

While co-inoculation with rhizobia and plant growth-promoting rhizobacteria (PGPR) can enhance soybean growth and nodulation, the interaction mechanisms between *Bacillus velezensis* 20507 and *Bradyrhizobium japonicum* USDA110 under varying nitrogen (N) supply levels (0–10 mmol/L) remain unclear. This study investigates how their synergistic interactions influence soybean nitrogen content per plant and molecular pathways.

**Methods:**

Soybean plants were co-inoculated with *B. velezensis* and *B. japonicum* across four N levels. Nodulation, plant growth, physiology, and N content were quantified. Transcriptome sequencing of soybean roots under N deficiency compared single and co-inoculation treatments. Flavonoids in B. velezensis fermentation broth were identified via mass spectrometry, and rutin’s regulatory effects on *B. japonicum* nodulation genes (NodD1/NodD2) were tested in coculture.

**Results:**

Co-inoculation significantly increased nodulation, biomass, and N content per plant compared to single inoculations across all N levels. Under N deficiency, co-inoculation induced 5,367 differentially expressed genes (DEGs), with Kyoto Encyclopedia of Genes and Genomes (KEGG) enrichment in phenylpropanoid (ko00940) and flavonoid biosynthesis (ko00941). *B. velezensis* produced 29 flavonoids and 4 isoflavonoids (including rutin). Rutin (5–10 mg/L) upregulated NodD1 and suppressed NodD2 in *B. japonicum*.

**Discussion:**

*B. velezensis* enhances *B. japonicum-soybean* symbiosis via flavonoid secretion, particularly rutin, which modulates nodulation gene expression. This metabiotic interaction improves soybean N assimilation and growth, even under low N conditions. The findings provide a foundation for designing composite inoculants to optimize soybean yield and nitrogen-use efficiency.

## 1 Introduction

The excessive use of chemical fertilizers in agricultural production has led to increasingly prominent problems such as soil fertility decline and environmental pollution. Microbial fertilizers have attracted widespread attention because they can enhance plant growth, crop yield, and quality, while reducing fertilizer use. *Bradyrhizobium japonicum* is a common microbial fertilizer that solubilizes potassium and phosphorus and promotes growth and symbiotic nitrogen fixation in soybeans (Nguyen et al., 2012; Afzal et al., 2010). *B. japonicum* provides nitrogen nutrition to its hosts and increases soybean yield through symbiotic nitrogen fixation in leguminous plants. *B. japonicum* secretes metabolites, such as organic acids, which can also chelate minerals, such as iron, phosphorus, and magnesium, making them one of the most valuable microbial resources in natural and agricultural systems (Argaw, 2012). Soybeans are important symbiotic nitrogen-fixing crops, with rhizobial nitrogen fixation supplying 50–90% of the nitrogen required for soybean growth (Bai et al., 2014). Rhizobial inoculation is widely used in many soybean-producing countries, including the United States, Brazil, and Argentina (Wu et al., 2017). However, the low survival rate of *B. japonicum* under environmental stress conditions, which requires repeated inoculation, limits its practical application (Armendariz et al., 2019). The unstable effects of inoculation have led to the development of optimized single strains (Melissa et al., 2022; Melchiorre et al., 2011), as well as multi-strain and multi-functional inoculants (Alemneh et al., 2020; Juge et al., 2012).

Combining *B. japonicum* with plant growth-promoting rhizobacterial (PGPR) strains is conducive to symbiotic nitrogen fixation and growth promotion (Alemneh et al., 2020; Mary et al., 2012), and soybean nodulation and nitrogen fixation can be enhanced by co-inoculation with other PGPR via various mechanisms (Zeffa et al., 2020; Majeed et al., 2015; Fox et al., 2011; Vessey, 2003). The potential mechanism of PGPR co-inoculation promoting the growth of leguminous plants mainly focuses on the following aspects: (1) PGPRs produce auxin, which can promote the growth and development of plant roots and improve the absorption capacity of nutrients and water (Kuan et al., 2016; Majeed et al., 2015; Shao et al., 2015); (2) PGPRs themself are capable of biological nitrogen fixing and provide essential ammonia for plant growth (Adedayo and Babalola, 2023; Sharma et al., 2022; Sun et al., 2022; Alemneh et al., 2020; Kuan et al., 2016; Majeed et al., 2015; Babu et al., 2015); (3) PGPRs can dissolve inorganic phosphate, providing more phosphorus for plant growth (Majeed et al., 2015); (4) PGPR can promote symbiosis between leguminous plants and nitrogen-fixing microorganisms, which is beneficial for the formation and development of plant nodules and enhances the ability of plants to fix nitrogen (Zeffa et al., 2020; Alemneh et al., 2020; Miransari, 2014; Mary et al., 2012; Fox et al., 2011; Vessey, 2003). Though synergistic effects of dual microbial inoculation involving arbuscular mycorrhizal (AM) fungi and free-living diazotrophs demonstrate significant enhancement in plant productivity through improved biomass accumulation and agricultural yield (Kasanke et al., 2024), To date, the mechanism by which PGPRs promote symbiosis between leguminous plants and nitrogen-fixing microorganisms has not yet been fully elucidated.

*Bacillus velezensis*, a spore-forming strain, is widely distributed in nature and used in agricultural production. Its ease of isolation and cultivation, rapid growth, and stress resistance make it a suitable inoculum (Cheng et al., 2024). It also promotes the growth of wheat, beets, carrots, peppers, potatoes, radishes (Meng et al., 2016), cucumbers (Sun et al., 2022), and soybean (Kondo et al., 2023). *B. velezensis* BAC03 promotes the growth of numerous plants under greenhouse conditions. Some studies have suggested that the mechanism by which this strain promotes plant growth may be related to its ability to synthesize auxins and ACC deaminase (1-aminocyclopropane-1-carboxylate deaminase) (Meng et al., 2016). *B. velezensis* SQR9 can directly promote the growth of cucumbers by producing auxins, volatile compounds acetoin and 2,3-butanediol, and extracellular phytases (Shao et al., 2015). There was a syntrophic cooperative relationship between the PGPR *B. velezensis* SQR9 and the indigenous *Pseudomonas stutzeri* in the cucumber rhizosphere. These two stains exchange metabolites and there was also a phenomenon of metabolic facilitation. Biofilm matrix components from *Bacillus* play an important role in this interaction, and the formation of a consortium is beneficial for promoting plant growth (Sun et al., 2022). *B. velezensis* S141 can promote the growth and nodulation of soybean plants, as well as the symbiosis between soybean and *B. diazoefficiency* USDA110. It is speculated that the main mechanism of action is that S141 can produce β-glucosidase, efficiently hydrolyzed isoflavone glucosides produced by soybean roots into isoflavone aglycones, and promote soybean nodulation. However, it is not clear which β-glucosidases are responsible for hydrolyzing isoflavone glucosides (Kondo et al., 2023).

*B. velezensis* exerts significant modulatory effects on indigenous soil microbial communities through competitive colonization dynamics and bioactive metabolite exchange. Spatial–temporal analyses reveal that *B. velezensis* FH-1 enhances early-stage seedling vigor in rice by reprogramming microbial community structure and functional profiles at critical developmental interfaces (Dai et al., 2024). Notably, *B. velezensis* SQR9 demonstrates dual functional capacities: direct plant growth promotion and metabolic mediation that selectively enriches native *P. stutzeri* populations. This interspecies synergy facilitates stable polymicrobial biofilm formation in the rhizosphere niche, establishing a persistent plant-beneficial consortium that significantly outperforms monoculture systems in cucumber cultivation trials (Sun et al., 2022; Chen et al., 2016). Mechanistic investigations of this syntrophic relationship reveal coordinated cellular protection mechanisms and enhanced Na^+^ homeostasis regulation under saline stress, providing the scientific basis for developing next-generation biofertilizers combining these microbial partners.

While extensive studies have documented individual applications of *B. velezensis* and *B. japonicum* in agricultural systems, critical knowledge gaps persist regarding their combinatorial effects on legume symbiosis regulation. Particularly, the mechanistic interplay between these microorganisms in modulating soybean growth-nodulation dynamics under varying nitrogen regimes remains poorly characterized—a significant oversight given the well-documented nitrogen-mediated suppression of diazotrophic activity (Saito et al., 2014). Our experimental design systematically evaluates single and co-inoculation strategies across nitrogen gradients, employing multi-omics approaches to: (1) quantify phenotypic responses in seedling growth and nodulation efficiency, (2) map root transcriptome reprogramming under nitrogen limitation, and (3) characterize metabolic cross-talk through bacterial exometabolite profiling. Crucially, we establish flavonoid-mediated regulation of *nod* gene expression as a key mechanism underlying microbial synergism. These findings collectively provide a robust theoretical framework for engineering advanced soybean inoculants and optimizing microbial consortia in sustainable fertilization practices.

## 2 Materials and methods

### 2.1 Microbial strains, culture, growth, and interaction

The experimental strain *B. velezensis* 20507 (CGMCC deposit No. 20507) was isolated and maintained in axenic culture in our laboratory, while *B. japonicum* USDA110 was procured from Prof. Xuelu Wang (Zhengzhou University, China). Both strains were cultivated under standardized conditions (28°C, 200 rpm orbital shaking) using selective media: PDA (Potato Dextrose Agar) for *B. velezensis* and TY (Tryptone Yeast extract) agar for *B. japonicum*. Bacterial biomass was harvested via centrifugation (5,200 × g, 20 min, 4°C) followed by three sequential deionized water washing cycles. To delineate nitrogen-dependent microbial interactions, a factorial design encompassing four nitrogen regimes (N0: 0 mM, N2: 2 mM, N5: 5 mM, N10: 10 mM Hoagland’s solution) and three inoculation strategies (monocultures: 10 μL of *B. velezensis* [Bv] or *B. japonicum* [Bj] at 1 × 10^9^ CFU/mL; co-culture [BB]: 5 μL Bv + 5 μL Bj) was implemented. Standardized cell suspensions (OD_600_ = 0.4) were inoculated into 400 μL culture systems (10 μL bacterial suspension +390 μL medium), with growth kinetics monitored using a Bioscreen C system (Lab Systems, Finland) under controlled parameters (28°C, 180 rpm, 7-day duration, OD_600_ measurements at 2-h intervals, *n* = 5 replicates). Concurrently, dual-culture assays on PDA plates inoculated with 20 μL aliquots of logarithmic-phase cultures (1 × 10^9^ CFU/mL per strain, 2.0 cm separation distance) facilitated spatial interaction analysis, with phenotypic documentation at 24-h intervals under standard incubation (28°C).

### 2.2 Plant growth trials

Pot experiments were conducted in controlled-environment chambers (School of Life Sciences, Jilin Normal University) under optimized growth parameters: 28°C/22°C Day/night thermoperiod, 70% relative humidity, and 12-h photoperiod with 400 μmol·m^−2^·s^−1^ photosynthetic photon flux density (LED illumination). *Glycine max* cv. Williams82 seeds underwent surface sterilization through sequential washing protocols: 5-min tap water cleansing followed by three 3-min sterile water rinses, culminating in 12-h imbibition at 28–30°C for synchronized germination. Germinated propagules were transplanted into 4.4-L horticultural pots (12.7 cm top Ø × 11.4 cm height) containing vermiculite substrate irrigated with nitrogen-gradient Hoagland solutions (0, 2, 5, 10 mM NO₃^−^; designated N0-N10). A full-factorial design incorporating four nitrogen levels and four inoculation treatments [CK: autoclaved control; Bv: *B. velezensis* 20507 (3 mL, 1 × 10^9^ CFU·mL^−1^); Bj: *B. japonicum* USDA110 (3 mL, 1 × 10^9^ CFU·mL^−1^); BB: 1:1 Bv:Bj co-inoculation] generated 16 experimental groups, each comprising three biological replicates (*n* = 8 seedlings/replicate). For experimental controls, sterilized inoculum (121°C, 20 min) replaced live bacterial suspensions. Physiological assessments at 30 days post-inoculation included: (1) chlorophyll quantification via SPAD-502PLUS meter (Konica Minolta, Japan), (2) photosynthetic gas exchange parameters measured by LI-6400XT portable photosynthesis system (Li-Cor Biosciences, United States) under standardized conditions (28°C leaf temperature, 400 μmol·mol^−1^ CO₂, 400 μmol·m^−2^·s^−1^ irradiance) during morning hours (09:00–12:00), and (3) morphometric analyses encompassing plant height, stem diameter (digital caliper), leaf area (YMJ-B area meter, Top Yunnong Tech, China), and biomass partitioning (aboveground tissues, root systems excluding nodules, and nodular structures). Tissue nitrogen content was determined through sulfuric acid-hydrogen peroxide digestion followed by automated Kjeldahl analysis (K-360, Büchi Labortechnik, Switzerland).

### 2.3 Transcriptome sequencing and enrichment analysis of differentially expressed genes

After 30 days of cultivation, roots with seedling nodules were sampled and sent to Sangon Biotech (Shanghai) Co., Ltd. for RNA extraction, sequencing on an Illumina HiSeq4000 PE150 platform, and bioinformatic analysis. The obtained raw transcriptome sequencing data were stored in SRA^1^ under the accession number PRJNA1152285. Clean data were aligned to the soybean reference genome using HISAT2 v.2.1.0 (Kim et al., 2015), and FastQC v0.11.2 was used to visually evaluate the sequencing data. StringTie v1.3.3b (Pertea et al., 2015) was used to assemble the mapped sequences onto the genome and evaluate gene expression levels. DESeq2 v1.12.4 was used to identify differentially expressed genes (DEGs) using the criteria of |Log_2_Fold Change| > 1.00 and false discovery rate (FDR) < 0.05. Genegene ontology and Kyoto Encyclopedia of Genes and Genomes enrichment analyses were performed using topGO v2.24.0, clusterProfiler v3.0.5, and an adjusted *Q*-value < 0.05, which was used as the criterion to judge the significantly enriched GO terms and KEGG pathways.

### 2.4 Metabolite extraction, detection, and data processing

*B. velezensis* 20507 was cultured for 3 days in PDA. The bacterial cells were then collected by centrifugation at 5,200 × g for 20 min at 4°C. The supernatant fermentation broth sample was freeze-dried, and 25 mg of solid sample was dissolved in 1,000 μL of extraction solution (methanol: acetonitrile: water = 2:2:1, (v/v/v)) containing an isotope-labeled internal standard. Vortex mixing was performed for 30 s, after which the solution was transferred to an ice-water bath and sonicated for 5 min. This step was repeated three times. After standing at −40°C for 1 h, the solution was filtered through a 0.22 μm filter membrane, and the filtrate was collected for detection. Vanquish (Thermo Fisher Scientific) ultra-high performance liquid chromatography was used to separate the target compound using a Phenomenex Kinetex C18 (2.1 mm × 50 mm, 2.6 μm) liquid chromatography column. The A phase of liquid chromatography was an aqueous phase containing 0.01% acetic acid and the B phase was isopropanol: acetonitrile (1:1, v/v). Column temperature: 25°C; sample tray temperature: 4°C; injection volume: 2 μL. The Orbitrap Exploris 120 mass spectrometer could collect primary and secondary mass spectrometry data under the control of a control software (Xcalibur, version 4.4, Thermo). The detailed parameters were as follows: sheath gas flow rate, 50 Arb; Aux gas flow rate, 15 Arb, Capillary temperature, 320°C; Sweep Gas, 1 arb; vaporizer temperature, 350°C. Full ms resolution: 60000, MS/MS resolution: 15000, Collision energy: SNCE 20/30/40, Spray Voltage: 3.8 kV (positive) or − 3.4 kV (negative). After converting the raw data into the mzXML format using ProteoWizard software, metabolite identification was performed using the R package (Zhou et al., 2022), and the database used was BiotreeDB (V3.0).

### 2.5 qRT-PCR

To investigate the regulatory effects of rutin (RU) and *B. velezensis* on *nodD1/nodD2* expression in *B. japonicum* USDA110, six experimental treatments were established in nitrogen-free Hoagland’s solution: (i) blank control (CK); (ii) *B. velezensis* monoculture (Bv); (iii–vi) RU gradient concentrations (5, 10, 20, 40 mg/L designated RU5-RU40). Pharmaceutical-grade rutin (≥98% purity, Sigma-Aldrich, United States) was dissolved in DMSO (0.1% final concentration). Co-inoculation treatments (BB) received both *B. velezensis* (1 × 10^8^ CFU/mL) and *B. japonicum* USDA110 (1 × 10^9^ CFU/mL) in 400 mL culture systems. Bacterial cells were harvested after 48-h incubation via centrifugation (5,000 × g, 10 min), followed by RNA extraction using the EASYspin Plus Kit (Aidibo Biotechnology, China). Quantitative reverse transcription PCR (qRT-PCR) was conducted on an AriaMx system (Agilent Technologies, United States) with 10 μL reactions under standardized cycling parameters: initial denaturation at 95°C (2 min); 40 cycles of 95°C (15 s), 60°C (15 s), 72°C (20 s). The 16S rRNA (USDA110) and β-actin (plant samples) served as endogenous controls for microbial and plant gene quantification, respectively. Twelve differentially expressed genes (DEGs) from RNA-seq comparisons (BB vs. Bj) were validated through this protocol. Relative gene expression was calculated via the 2^−ΔΔCt^ method (Schmittgen and Livak, 2008) with triplicate technical replicates. Primer sequences are cataloged in Supplementary Table S1.

### 2.6 Statistical analysis

Variance analysis was performed using two-way ANOVA (main factors of four N concentration treatments and four microbial inoculation treatments) in SPSS (Statistical Package for the Social Sciences) software version 26, with a *t*-test evaluating statistically significant differences at the 5% level.

## 3 Results

### 3.1 Effect of *Bacillus velezensis*, *Bradyrhizobium japonicum* inoculation and nitrogen concentration on seedling growth

**Figure 1:**
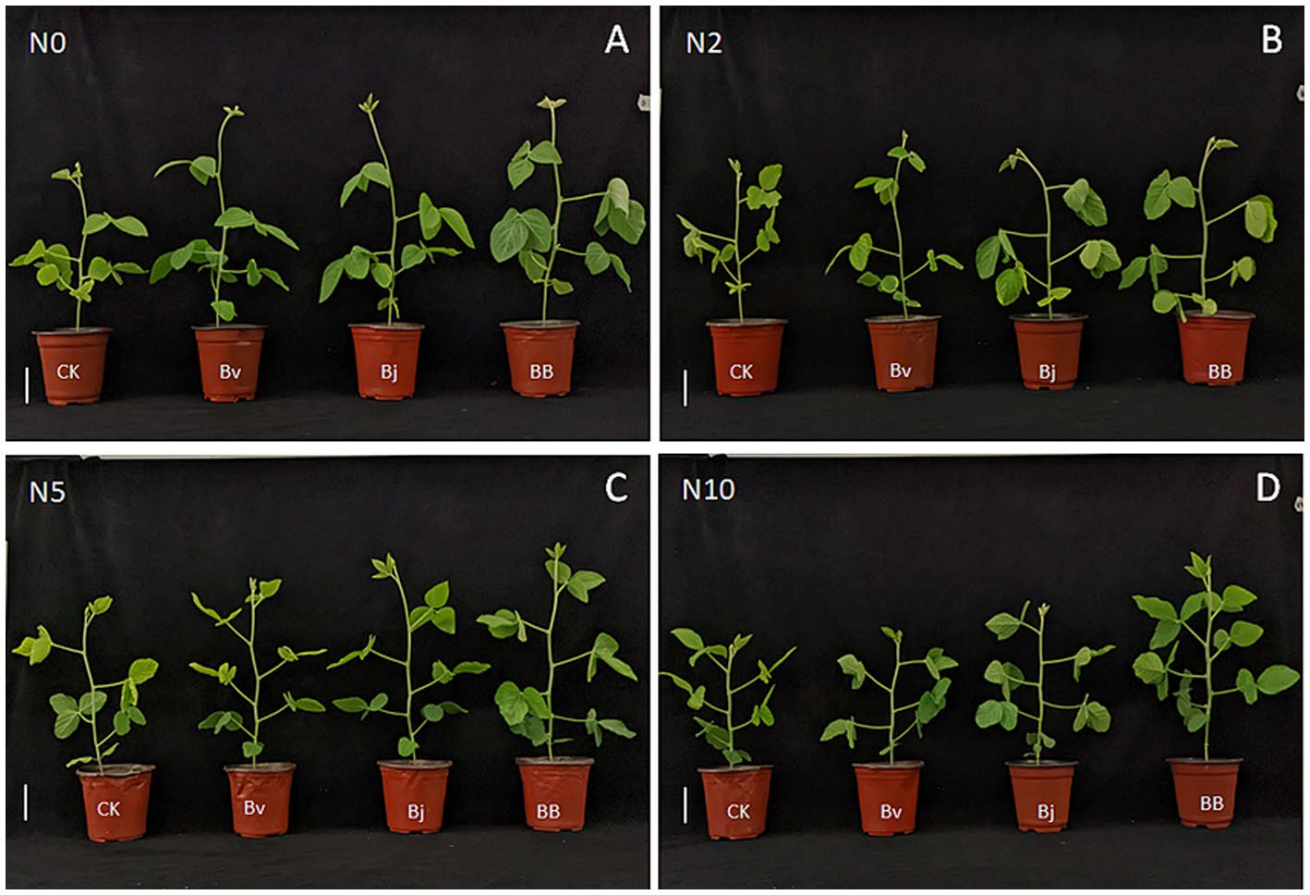
Phenotypic responses of soybean seedlings to nitrogen gradients and bacterial inoculation treatments. **(A–D)** Representative images under 0, 2, 5, and 10 mM nitrogen concentrations with four treatments: CK (control), Bv (*Bacillus velezensis*), Bj (*Bradyrhizobium japonicum* USDA110), and BB (Bv + Bj co-inoculation). Scale bars: 8.0 cm. Plants were cultivated in vermiculite for 30 days.

The nitrogen concentration gradient in the culture medium and microbial inoculation significantly affected the growth of soybean seedlings (Figure 1). Compared with the control under the same nitrogen concentration, the seedling heights of the Bv, Bj, and BB treatments were higher under N0 conditions (Figure 1A). These results suggest that compared to the control, the Bv, Bj, and BB treatments enhanced soybean seedling growth. Moreover, among the different inoculation treatments, the BB treatment resulted in the most pronounced growth improvement. Similarly, under N2 (Figure 1B), N5 (Figure 1C), and N10 (Figure 1D) conditions, seedlings treated with Bv, Bj, or BB showed better growth than the control, with the BB treatment consistently achieving the highest growth performance. Collectively, these findings indicate that separate inoculation of *B. velezensis* or *B. japonicum* promotes soybean growth, while combined inoculation (BB) synergistically enhances seedling growth compared to single-strain applications.

### 3.2 Effect of nitrogen concentrations on growth curves of *Bacillus velezensis* and *Bradyrhizobium japonicum*

**Figure 2:**
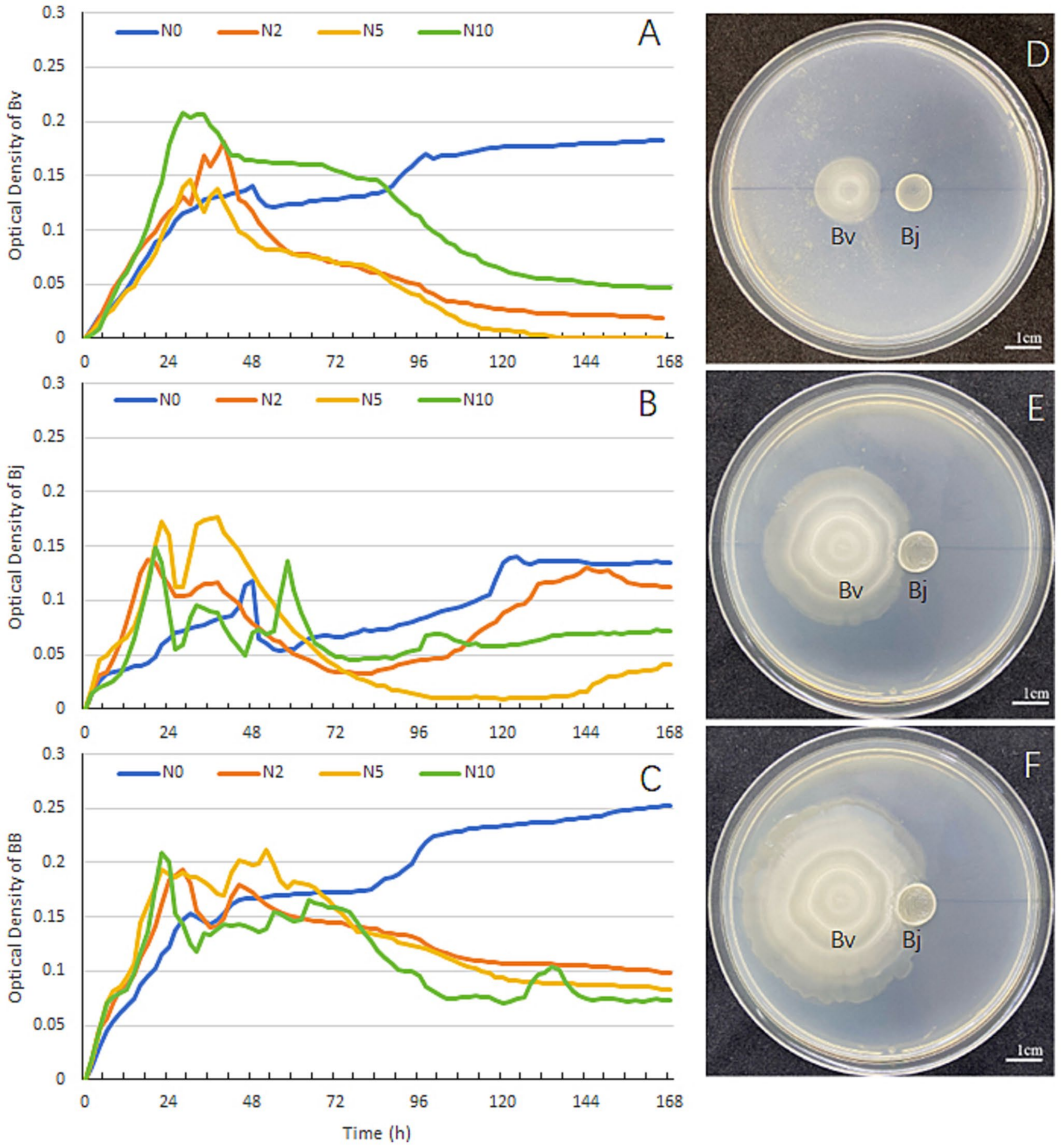
*In vitro* growth dynamics and spatial interactions between *B. velezensis* and *B. japonicum* USDA110. Growth curves of **(A)** *B. velezensis*, **(B)** *B. japonicum*, and **(C)** co-culture under four nitrogen regimes (N0-N10: 0–10 mM). **(D–F)** Plate confrontation assay at 24, 48, and 72 h post-inoculation. Cultures were maintained for 7 days at 28°C with continuous shaking (200 rpm).

Soybean seedlings were nourished with Hoagland’s solution containing graded nitrogen levels (N0, N2, N5, N10). *B. velezensis* and *B. japonicum* were inoculated either singly or in combination in the respective nitrogen media, and their growth curves were compared. For *B. velezensis*, the OD values in N2, N5, and N10 treatments exhibited an initial increase followed by a decline, whereas in N0, the OD value rose steadily, showing divergent trends among treatments (Figure 2A). After 168 h, the highest OD value for *B. velezensis* was observed in N0, followed by N10, while N2 and N5 had the lowest values (Figure 2A). *B. japonicum* displayed a similar pattern, with the highest OD value in N0 and the lowest in N5 and N10 after 168 h (Figure 2B). In co-inoculation experiments, the OD value in N0 was markedly higher than in other treatments at 168 h (Figure 2C). Specifically, under nitrogen-deficient conditions (N0), the OD value of the mixed inoculation reached 0.25 (Figure 2C), exceeding the values for *B. velezensis* (0.18; Figure 2A) and *B. japonicum* (0.13; Figure 2B) in monoculture. Plate confrontation assays (Figures 2D–F) revealed no antagonism between the two strains. Based on the observed metabolic interdependence, we propose that their interaction represents metabiosis—a relationship where organisms benefit mutually through metabolic activities. In summary, both strains exhibited higher proliferation rates in nitrogen-deficient media than in nitrogen-supplemented media, and their interaction was characterized by metabiosis.

### 3.3 Effects of nitrogen concentrations and strain inoculation on height, stem diameter, leaf area, and SPAD of soybean seedlings

**Figure 3:**
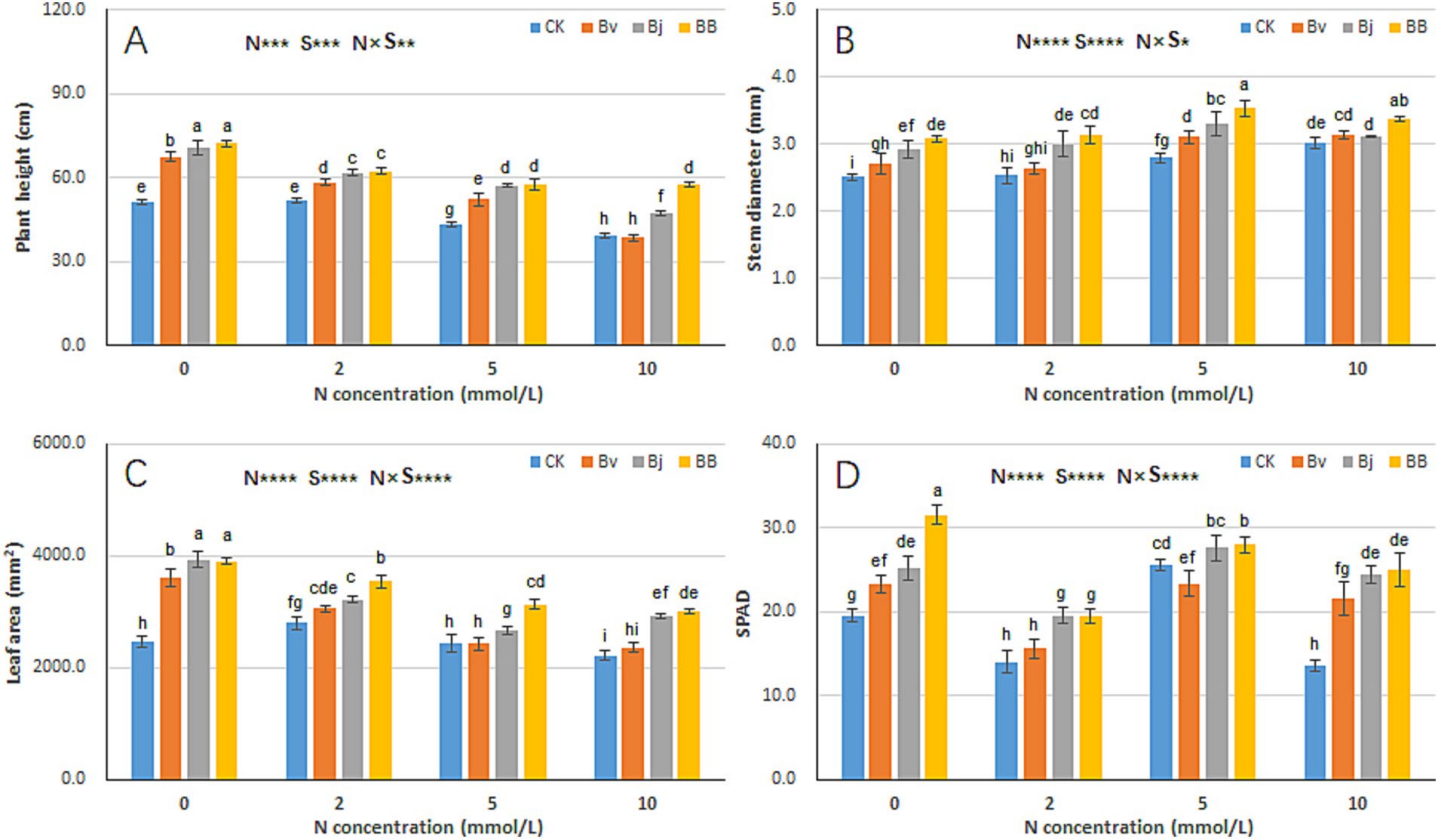
Nitrogen-inoculation interactions on soybean growth parameters. **(A)** Plant height, **(B)** stem diameter, **(C)** leaf area, and **(D)** SPAD values (Soil Plant Analysis Development). N: nitrogen levels (0–10 mM); S: treatments (CK, Bv, Bj, BB). Data represent mean ± SD (*n* = 12). Distinct lowercase letters denote significant differences (*t*-test, *p* < 0.05; ** < 0.01; *** < 0.001; **** < 0.0001).

Nitrogen concentration (N), strain inoculation (S), and N × S interaction significantly influenced plant height, stem diameter, leaf area, and SPAD (Figure 3). Increasing nitrogen levels led to reduced plant height (Figure 3A) but increased stem diameter (Figure 3B), resulting in more robust seedlings. Under the same nitrogen concentration, Bj and Bv treatments increased both plant height and stem diameter, while BB further enhanced these parameters compared to CK, Bj, and Bv (Figures 3A,B). Similarly, leaf area declined with increasing nitrogen levels under N2, N5, and N10 (Figure 3C), yet BB treatment maximized leaf area across all nitrogen conditions (Figure 3C). SPAD values were significantly higher in Bj and BB treatments than in CK under the same nitrogen levels (Figure 3D). Overall, the BB treatment achieved the highest values for all measured traits (plant height, stem diameter, leaf area, SPAD) and most effectively promoted soybean seedling growth.

### 3.4 Effects of nitrogen concentrations and strain inoculation on plant weight and root nodule weight of soybean seedlings

**Figure 4:**
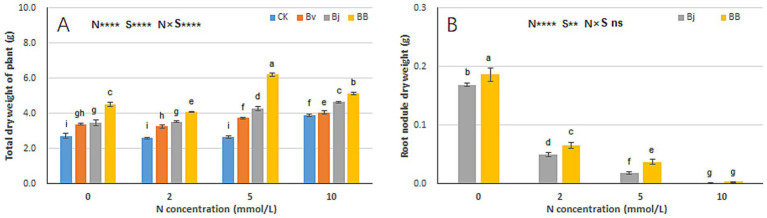
Biomass allocation patterns under experimental treatments. **(A)** Whole-plant dry weight, **(B)** nodule dry weight. Statistical conventions as in Figure 3.

Nitrogen (N), strain inoculation (S), and N × S interaction significantly affected total plant dry weight (Figure 4A). Additionally, N and S influenced root nodule dry weight (Figure 4B). Under the same nitrogen concentration, Bj and BB treatments yielded higher total plant weights than CK, with BB consistently outperforming other treatments (Figure 4A). In pot experiments, root nodules formed exclusively in Bj and BB treatments (inoculated with *B. japonicum*), whereas CK and Bv (lacking *B. japonicum*) produced no nodules. Root nodule weight was inversely correlated with medium nitrogen content. At N0, N2, and N5, BB treatment generated significantly heavier nodules than Bj (Figure 4B), suggesting that *B. velezensis* facilitates *B. japonicum* infection and nodulation in soybean roots.

### 3.5 Effects of nitrogen concentrations and strain inoculation on photosynthetic characteristics of soybean seedlings

**Figure 5:**
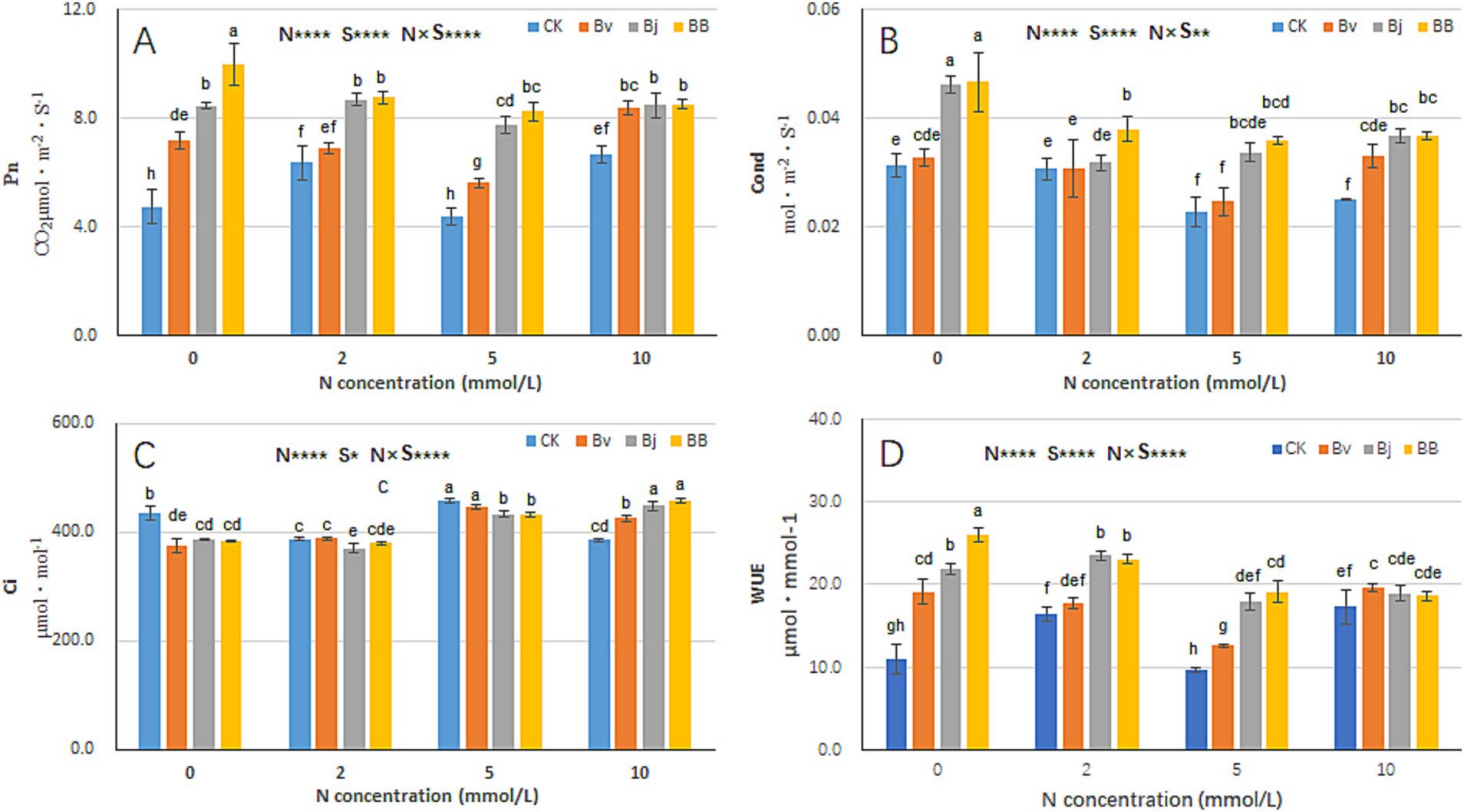
Photosynthetic performance under combinatorial treatments. **(A)** Net photosynthetic rate (Pn), **(B)** stomatal conductance (Cond), **(C)** intercellular CO_2_ concentration (C_i_), **(D)** water-use efficiency (WUE). Data represent mean ± SD (*n* = 6). Statistical analysis as in Figure 3.

The photosynthetic characteristics of soybean seedlings under different nitrogen concentrations and inoculation conditions were analyzed. Nitrogen (N), strain inoculation (S), and N × S interaction significantly affected photosynthetic parameters and water use efficiency (WUE) (Figure 5). Under the same nitrogen concentration, net photosynthetic rate (Pn) values of Bj (*B. japonicum*), Bv (*B. velezensis*), and BB (*B. japonicum* + *B. velezensis*) treatments were higher than those of CK (control). Notably, BB treatment exhibited the highest Pn values, demonstrating superior enhancement of photosynthetic efficiency compared to single-strain treatments (Figure 5A). Under N0, N2, and N5 conditions, intercellular CO₂ concentration (Ci) values of BB were lower than those of CK, Bj, and Bv (Figure 5C), while stomatal conductance (Cond) and WUE of BB were consistently higher than other treatments (Figures 5B,D). These findings suggest that co-inoculation (BB) synergistically optimizes photosynthetic performance and water utilization in soybean seedlings.

### 3.6 Effects of nitrogen concentrations and strain inoculation on nitrogen content of soybean seedlings

**Figure 6:**
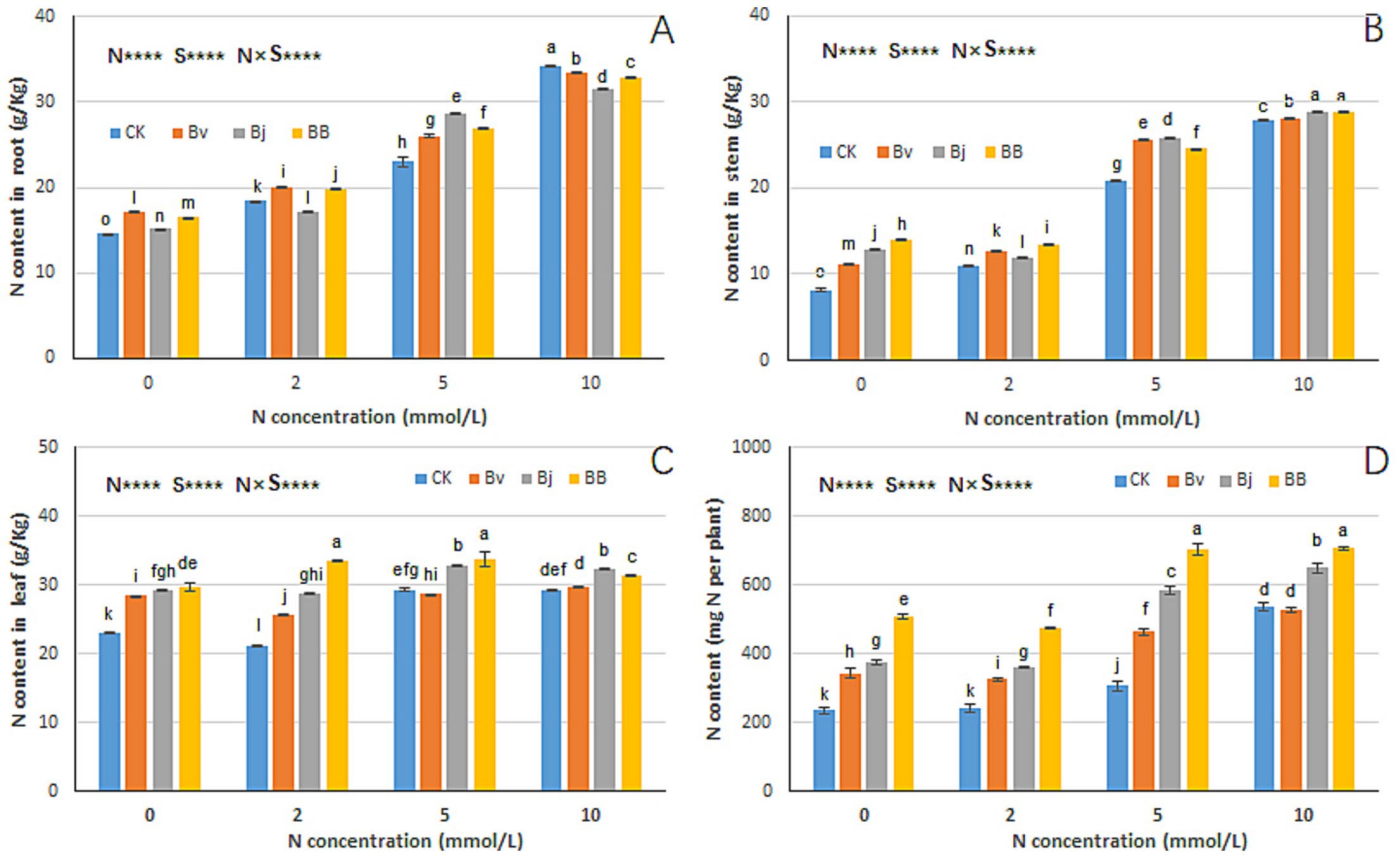
Nitrogen partitioning in plant tissues. Tissue-specific N content in **(A)** roots, **(B)** stems, **(C)** leaves, and **(D)** whole plants. Experimental conditions and statistical methods as in Figure 3.

Nitrogen (N), strain inoculation (S), and N × S interaction significantly influenced nitrogen content in roots, stems, leaves, and total plant nitrogen (Figure 6). Increasing nitrogen levels correlated with elevated stem and leaf nitrogen content (Figures 6A,B). Under the same nitrogen concentration, Bj and BB treatments accumulated more nitrogen in leaves and stems than CK. Specifically, under N0 conditions, BB treatment achieved the highest nitrogen content, significantly surpassing CK and Bj (Figures 6A–C). Total plant nitrogen content increased with nitrogen concentration (Figure 6D), with the following efficacy ranking across treatments: BB > Bj > Bv > CK under N0, N2, and N5 conditions. At N10, BB, and Bj maintained higher total nitrogen than CK and Bv, highlighting BB as the most effective strategy for enhancing nitrogen uptake efficiency.

### 3.7 Differentially expressed genes

Twelve root samples from the soybean seedlings were collected and sequenced, generating 603,274,676 raw reads. These reads covered a length of 90,491,201,400 bp (Supplementary Table S2). Low-quality reads were filtered, and 562, 043, and 316 clean reads were generated. Clean reads were mapped to the soybean reference genome, and 546,957,198 clean reads were aligned to the reference genome, accounting for 97.31% of the obtained clean reads (Supplementary Table S3). All DEGs in paired comparison of BB vs. Bj are listed in Supplementary Table S4.

**Figure 7:**
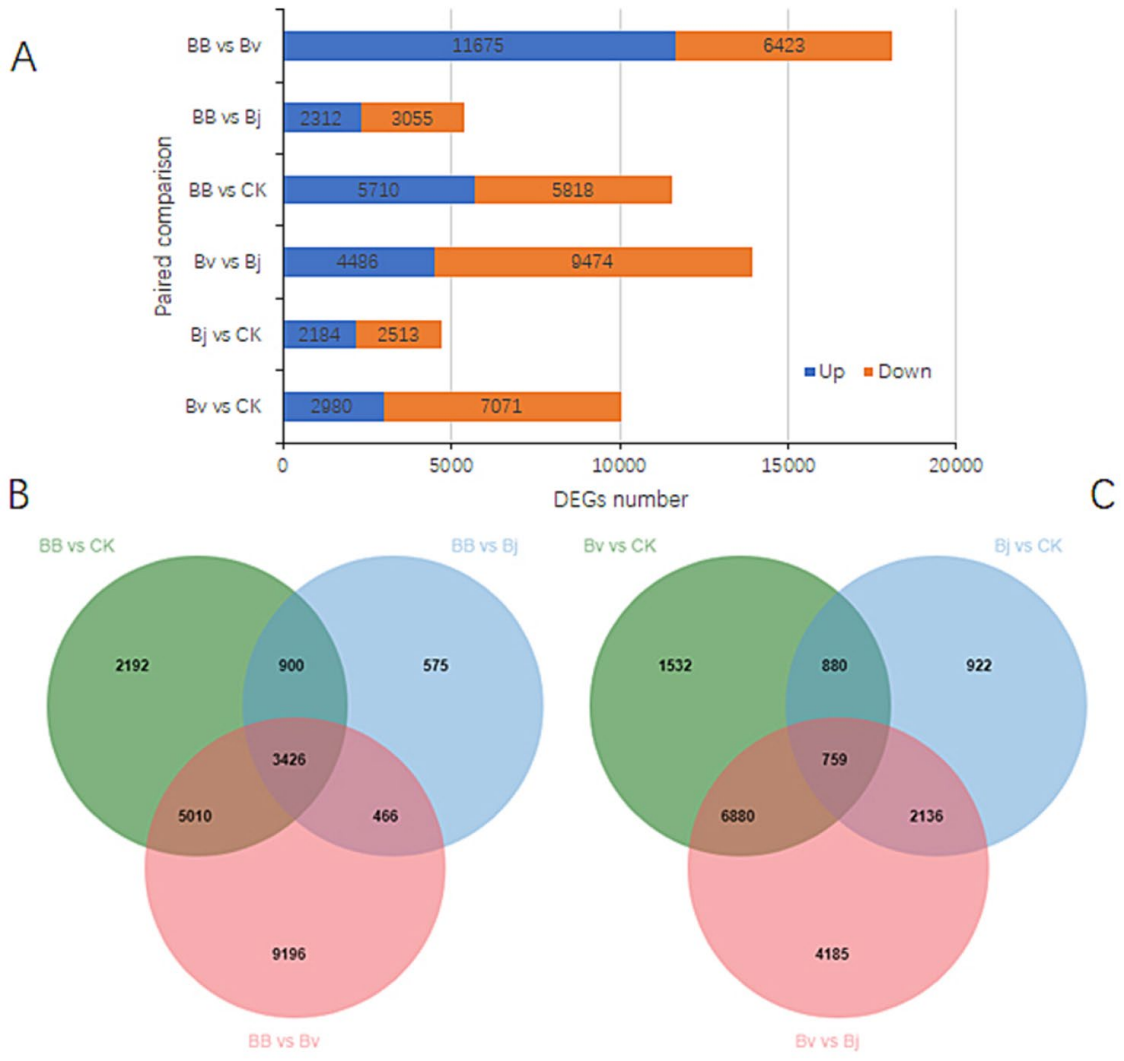
Differential gene expression profiling. **(A)** DEG counts across six comparisons. Venn diagrams of shared DEGs in **(B)** BB-centric comparisons and **(C)** pairwise monoculture contrasts. CK: control; BB: Bv + Bj co-inoculation.

There were 10,051 DEGs in the paired comparison of Bv vs. Ck, and the number of downregulated genes was 2.37 times that of upregulated genes. There were 4,697 DEGs between Bj and Ck, and the number of downregulated genes was relatively close to that of upregulated genes. There were 13,960 DEGs in Bj vs. Bv, and the number of downregulated genes was 2.11 times that of upregulated genes (Figure 7A). There were 18,098 DEGs in BB vs. Bv, and the number of upregulated genes far exceeded that of downregulated genes. There were 5,367 DEGs in the BB vs. Bj group, which was much fewer than the number of DEGs in the BB vs. Bv. There were 11,528 DEGs between BB and CK, and the numbers of upregulated and downregulated genes were similar (Figure 7B). The three comparisons generated 21,765 DEGs, and the common DEGs shared by the three groups accounted for 15.74% of all DEGs (Figure 7B). Among the three paired comparisons, BB vs. Bv produced the most DEGs, whereas BB vs. Bv produced fewer DEGs. In pairwise comparisons of Bv vs. CK and Bj vs. CK, there were more DEGs in Bv vs. CK. These results indicate that Bv significantly impacted the expression of soybean root genes, whereas Bj had a relatively small impact on the expression of root genes. In the three paired comparisons of Bv vs. CK, Bj vs. CK, and Bv vs. Bj, 17,294 DEGs were generated, with only 759 DEGs shared among the three, accounting for only 4.39% of all the DEGs (Figure 7C). Thus, there was a significant difference between the DEGs induced by Bv and Bj treatments.

### 3.8 GO clustering analysis of DEGs in BB vs. Bj

**Figure 8:**
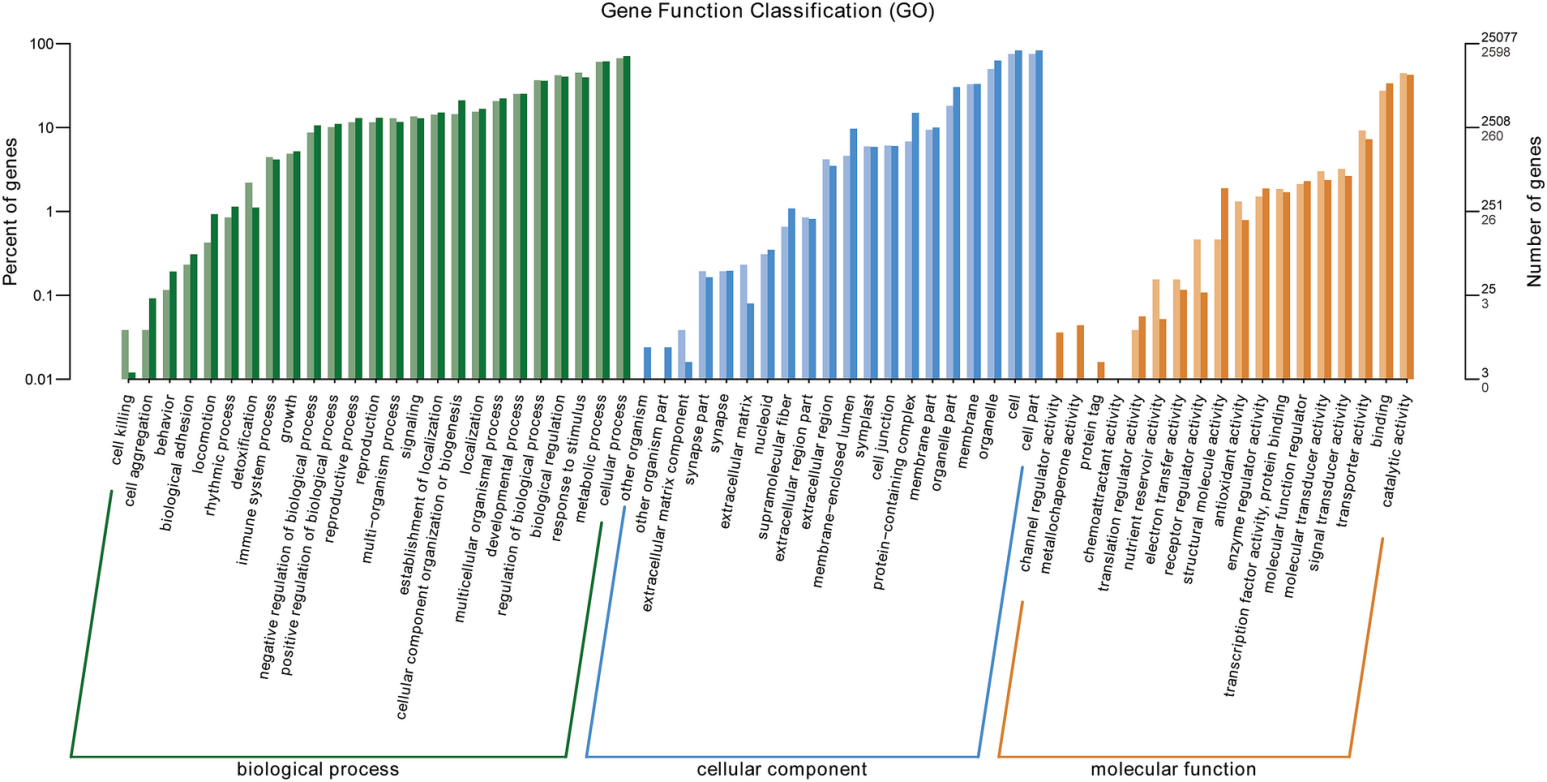
Gene ontology (GO) enrichment of BB vs. Bj DEGs. X-axis: GO terms; Y-axis: gene count/percentage.

The GO enrichment of all DEGs in BB vs. Bj comparison are listed in Supplementary Table S5. GO (Gene Ontology) clustering analysis of DEGs in BB vs. Bj was performed, and the results are shown in Figure 8. All DEGs were clustered into 63 GO Terms annotated to 25 biological processes, 20 cellular components, and 18 molecular functions. Catalytic activity (GO: 0003824) was the GO term with the most members in the molecular function category, with 1,150 DEGs, followed by binding (GO: 0005488) and transporter activity (GO: 0005215) with 711 and 239 DEGs, respectively. The GO term with the most members in the cellular component category was cell part (GO: 0044464), with 1,952 DEGs, followed by organelle (GO: 0043226) and membrane (GO: 0044425), with 1,289 and 851 DEGs, respectively. The GO term with the most members in the biological process category was cellular process (GO: 0009987), followed by metabolic process (GO: 0008152), and response to stimulus (GO:0050896). Based on these results, it is believed that co-inoculation with BB and Bj significantly impacted the expression of genes related to metabolism, response to stimulus, and substance transport in soybean seedling root cells compared with Bj treatment alone.

### 3.9 KEGG pathway enrichment analysis of significantly differentially expressed genes in paired comparison of BB vs. Bj

**Figure 9:**
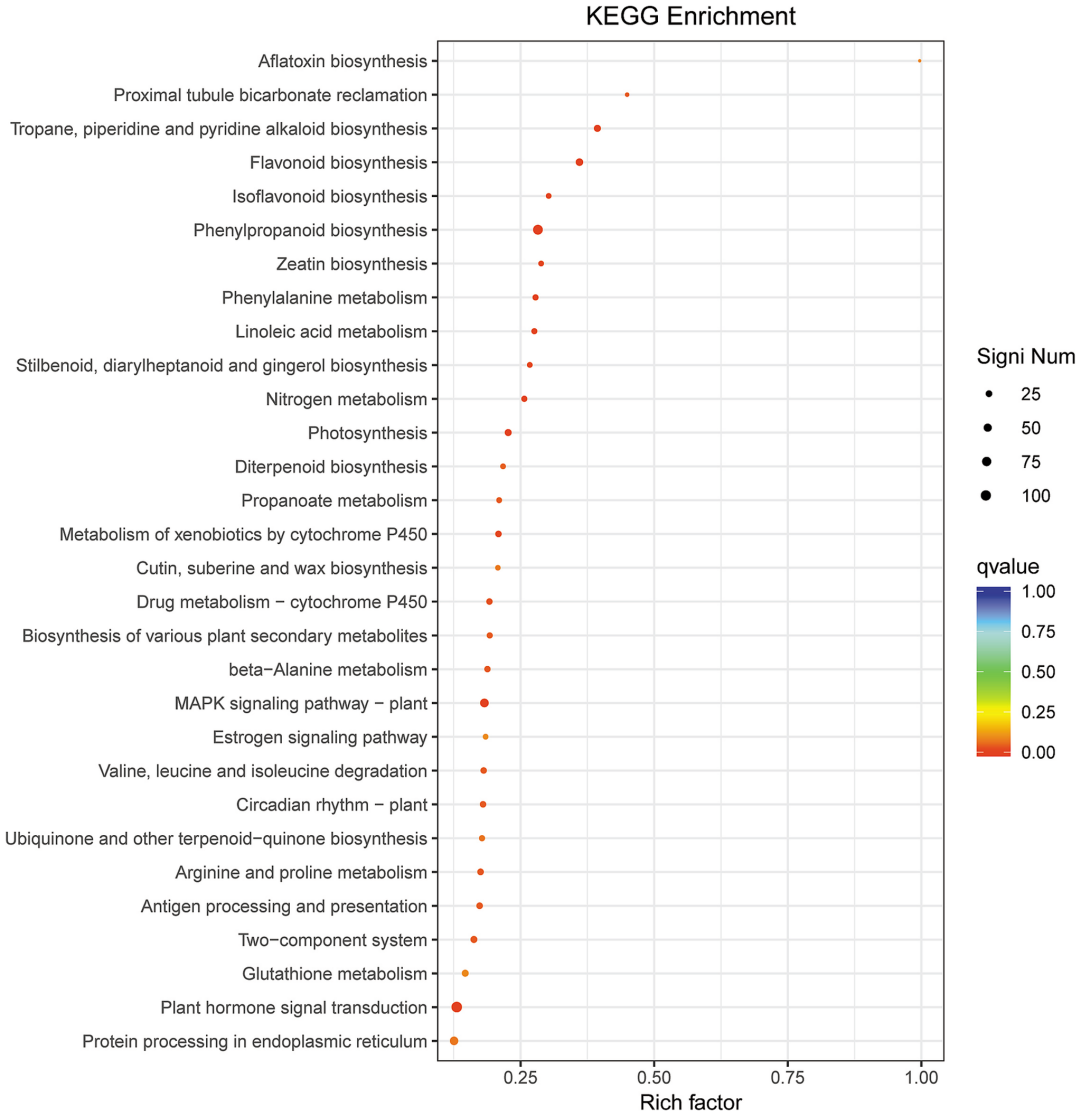
KEGG pathway analysis of BB vs. Bj DEGs. Dot plot visualization with rich factor (DEG proportion per pathway), *Q*-value (color gradient), and gene count (dot size). Red coloration indicates higher significance.

KEGG enrichment analysis revealed 46 significantly enriched pathways in the BB vs. Bj comparison (Supplementary Table S6; Figure 9), despite no pathways being enriched in Bv vs. Bj (13,960 DEGs), indicating divergent molecular mechanisms between *B. velezensis* (Bv) and *B. japonicum* (Bj). Top enriched pathways included: phenylpropanoid biosynthesis (ko00940), flavonoid biosynthesis (ko00941), MAPK signaling (ko04016), photosynthesis (ko00195), plant hormone transduction (ko04075), and nitrogen metabolism (ko00910), suggesting co-inoculation (BB) broadly regulates secondary metabolism, stress adaptation, and nutrient utilization compared to Bj alone.

### 3.10 Mass spectrometry identification of *Bacillus velezensis* 20507 fermentation broth

**Figure 10:**
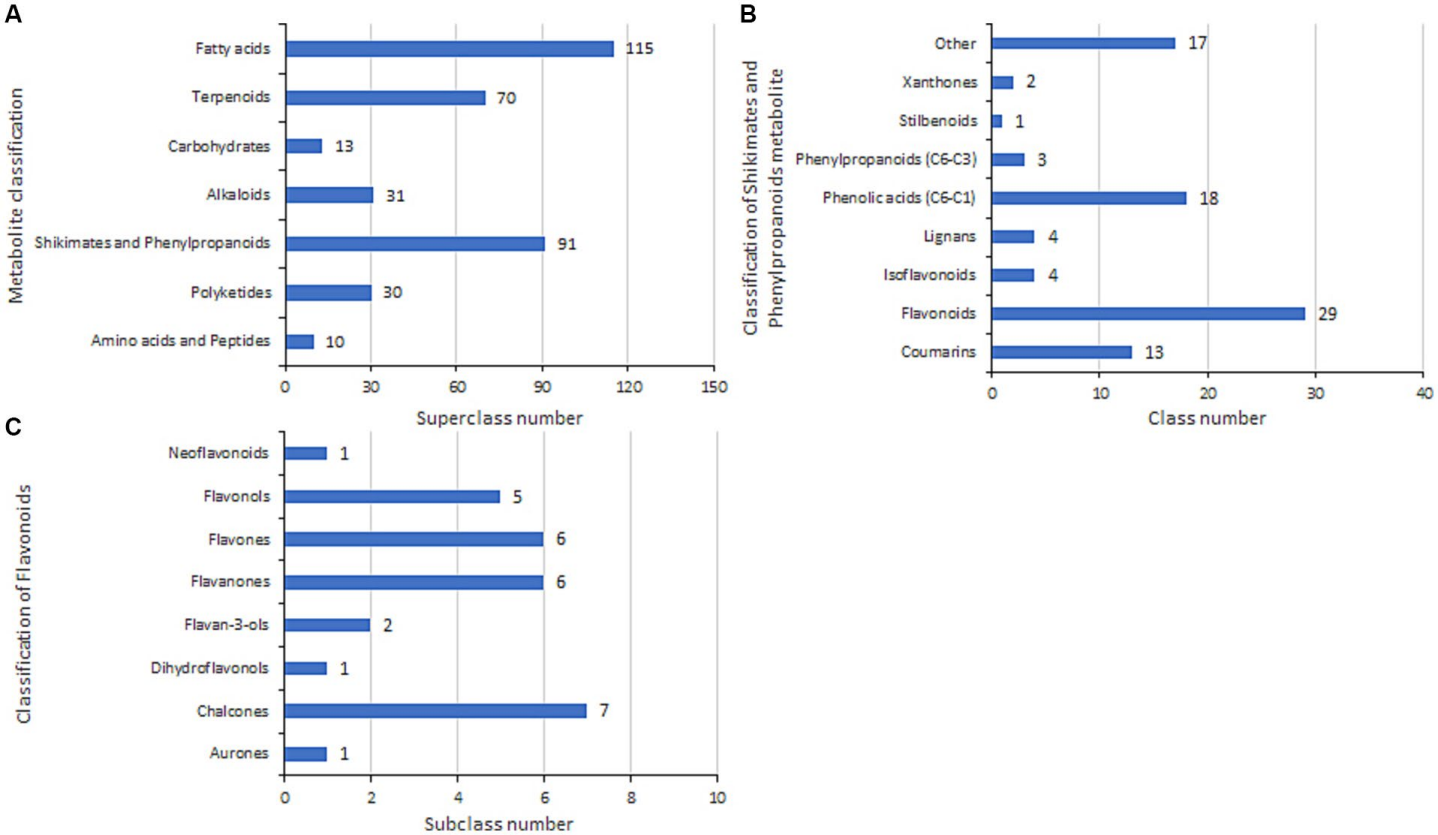
Metabolomic profiling of *B. velezensis* 20507 fermentation products. **(A)** Metabolite superclass distribution. **(B)** Shikimate-phenylpropanoid derivatives. **(C)** Flavonoid subclasses.

We sequenced and assembled the genome of *B. velezensis* 20507 and identified the chalcone synthase gene (tig00000001_pilon_281) (Supplementary Table S7), which is considered a key enzyme in flavonoid synthesis. Furthermore, to determine whether *B. velezensis* 20507 secretes flavonoids to promote the growth of USDA110 and soybean seedlings, metabolites were identified in the fermentation broth of *B. velezensis* 20507, and the identified compounds are listed in Supplementary Table S8. There were 390 metabolites in the broth of *B. velezensis* 20507, of which 360 belonged to seven superclasses (Figure 10A). The fatty acid superclass had the largest number of members, accounting for 29.49% of the total identified compounds, followed by shikimates and phenylpropanoids with 91 compounds accounting for 23.33% of the total. Further classification of shikimates and phenylpropanoids revealed that there were 29 flavonoid classes and four isoflavonoid classes, accounting for 31.87 and 4.40% of the total compounds in shikimates and phenylpropanoids, respectively (Figure 10B). The 29 flavonoid compounds were further divided into eight subclasses, including chalcones (e.g., 4-Hydroxydericin and Droloxifene), flavanones (e.g., Bavachinin and Glabrol), flavones (e.g., Hosloppin and Oroxindin), and flavonols (e.g., Rutin and Kaempferol) contained numerous compounds, totaling 24, accounting for 83% of flavonoid compounds (Figure 10C). In summary, total flavonoid/isoflavonoid content reached 8% of identified metabolites, supporting the role of *B. velezensis* in flavonoid-mediated symbiosis enhancement.

### 3.11 qRT-PCR of DEGs

**Figure 11:**
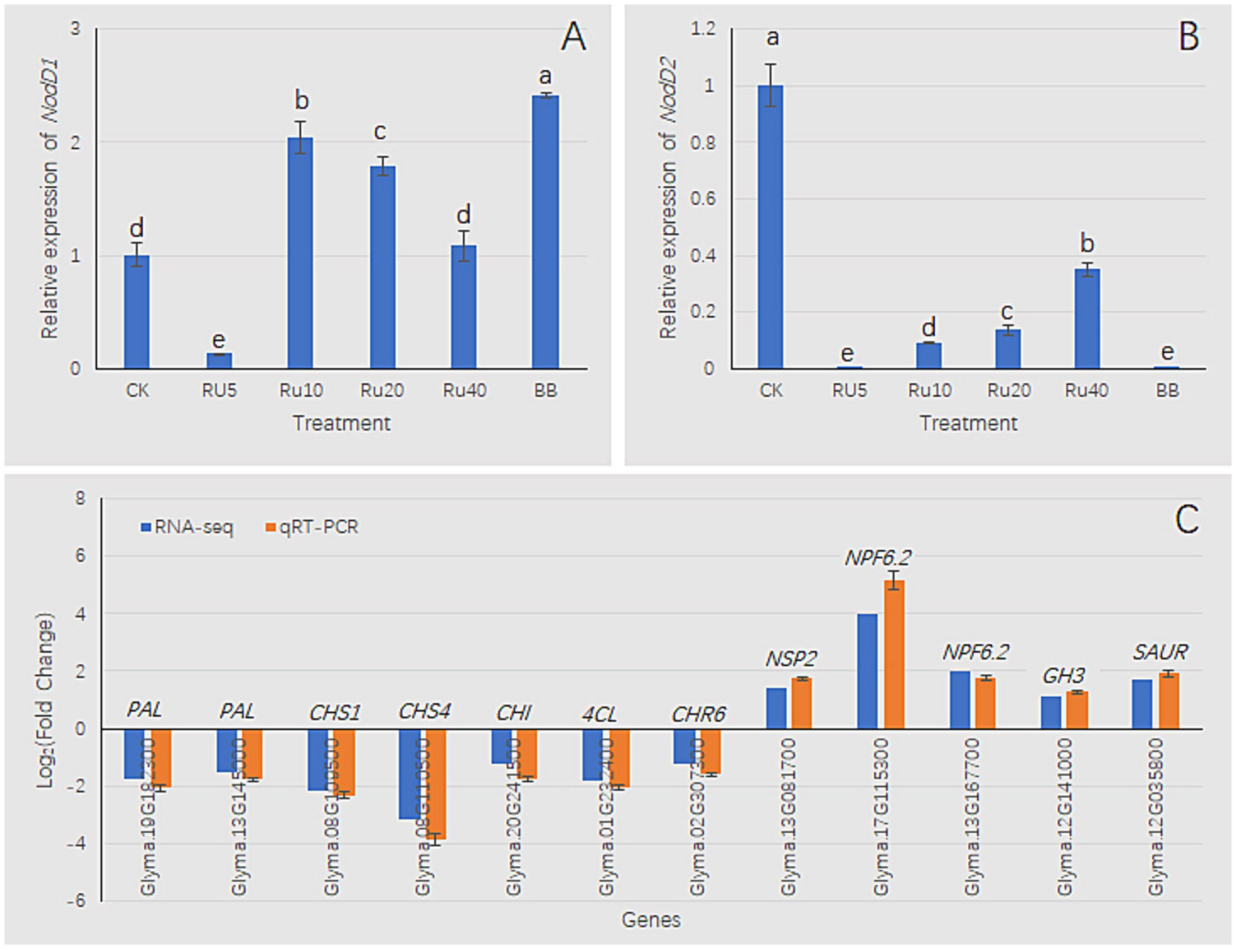
qRT-PCR analysis. **(A,B)** *nodD1/nodD2* expression in *B. japonicum* USDA110 under rutin (RU) gradients (5–40 mg/L) with/without Bv co-inoculation. **(C)** RNA-seq verification using 16S rRNA as endogenous control. Fold change calculated as BB/Bj ratio. Bars: mean ± SD (*n* = 3); lowercase letters indicate significance (LSD, *p* < 0.05). Enzyme abbreviations: PAL, phenylalanine ammonia lyase; CHS, chalcone synthase; CHI, chalcone isomerase; 4CL, 4-coumarate-CoA ligase; CHR6, chalcone reductase 6; NSP2, nodulation signaling pathway2; NPF6.2, nitrate transporter1/ peptide transporter family6.2; GH3, GRETCHEN HAGEN3; SAUR, small auxin-up RNA.

The addition of rutin to the culture medium and co-inoculation with *B. velezensis* had vital effects on the expression of *NodD1* and *NodD2*. Within the range of 0–10 mg/L rutin, the expression level of *NodD1* increased with increasing rutin concentrations. Within the range of 10–40 mg/L, the expression level of *NodD1* decreased with increasing rutin concentration (Figure 11A). Compared with the control, the three treatments (RU5, RU10, and BB) significantly increased the expression levels of *NodD1* (Figure 11A). Compared with the control, the other five treatments significantly reduced *NodD2* expression levels (Figure 11B). Thus, the trend of *NodD1* was opposite to that of *NodD2* (Figures 11A,B). For paired comparisons of BB and Bj, a set of DEGs was selected for qRT-PCR analysis. RNA-seq and qRT-PCR results showed that DEGs related to flavonoid synthesis, such as *PAL* (*phenylalanine ammonia lyase*), *CHS* (*chalcone synthase*), *CHI* (*chalcone isomerase*), *4CL* (*4-coumaric coenzyme A ligase*), and *CHR6* (*chalcone reductase6*), were downregulated; nitrogen transport-related genes *NSP2* (*nodulation signaling pathway2*) and *NPF6.2* (*nitrate transporter1/ peptide transporter family6.2*) were upregulated, and DEGs related to auxin signal transduction, *GH3* (*GRETCHEN HAGEN3*) and *SAUR* (*small auxin-up RNA*), were upregulated (Figure 11C). These results suggest that BB treatment may inhibit flavonoid synthesis, promote nitrogen transport, and alter auxin signaling in soybean seedlings. The trends in gene expression changes in RNA-seq and qRT-PCR were similar, indicating that the RNA-seq analysis results in this study were reliable.

## 4 Discussion

### 4.1 Co-inoculation of *Bacillus velezensis* and *Bradyrhizobium diazoefficiens* USDA110 promotes the growth and nodulation of soybean

Existing research has shown that PGPR are widely used in agriculture and have promising application prospects. PGPR can be used alone or combined with other microbial species to improve their application (Alemneh et al., 2020). Besides its own disease resistance and growth promotion, PGPR co-inoculated with rhizobia can also promote nodulation and nitrogen fixation in rhizobia and improve soil fertility and nutrient utilization efficiency through the synergistic effect of the two (Zeffa et al., 2020; Majeed et al., 2015; Fox et al., 2011; Vessey, 2003). The PGPR strains with these include *Azotobacter* (Abdiev et al., 2019), *Azospirillum* (Chibeba et al., 2015; Remans et al., 2008a), *Pseudomonas* (Nascimento et al., 2019), *Streptomyces* (Htwe et al., 2018), *Bacillus* (Korir et al., 2017), *Staphylococcus* (Prakamhang et al., 2015), and *Serratia* (Zahir et al., 2011). Juge et al. (2012) studied the effects of different inoculation methods of *Bradyrhizobium*, *Azospirillum*, and arbuscular mycorrhizae on soybean growth and found that all microbial combinations improved soybean root biomass. Argaw (2012) studied the effects of co-inoculation of *B. japonicum* and phosphate-solubilizing *Pseudomonas* spp. and traditional fertilizers on soybean nodulation, yield, and yield components, and found that co-inoculation could maximize soybean yield and quality. Mary et al. (2012) studied the effect of co-inoculation with *B. japonicum* and *Bacillus subtilis* on soybeans and found that co-inoculation could increase the number of nodules, dry weight of nodules, and biomass of soybeans. Co-inoculation with *P. mucilaginosus* and *B. japonicum* not only improved soybean quality and increased yield, but also increased soil fertility (Ma et al., 2015). Compared with single inoculation, co-inoculation with PGPR and USDA110 resulted in a significant increase in the number of root nodules, root nodule dry weight, and seed yield (Aung et al., 2013), and co-inoculation of *B. subtilis* or *Staphylococcus aureus* with USDA110 increased the nodule number and biological yield (Prakamhang et al., 2015). In this study, the plant height, stem diameter, biomass, and other indicators of soybean seedlings inoculated with *B. velezensis* and *B. japonicum* were significantly higher than those inoculated with *B. velezensis* and *B. japonicum* alone, indicating that the co-inoculation of *B. velezensis* and *B. japonicum* improved the growth of soybean seedlings, consistent with previous reports (Korir et al., 2017; Prakamhang et al., 2015). While the current study demonstrates *B. velezensis*’ rhizospheric competence, GFP-based spatial tracking coupled with confocal microscopy could further elucidate its microhabitat preferences and definitively rule out transient endophytic colonization. This study provides a new approach for the development of nitrogen-fixing bacterial fertilizers for soybean.

### 4.2 *Bacillus velezensis* secreted flavonoids to promote symbiosis between USDA110 and soybeans

During the process of mutual recognition between soybeans and rhizobia, transduction of biochemical signals is a prerequisite for establishing a symbiotic system (Nakei et al., 2022). Leguminous plant roots secrete flavonoids in N-deficient environments, such as flavonoids, which have chemotactic effects on rhizobia and can attract rhizobia to the soil and bind them to the roots of leguminous plants, stimulating their massive reproduction (Sharma et al., 2021). Host-specific flavonoids interact with NodD proteins in rhizobia and induce *Nod* expression (Liu and Murray, 2016; Mario et al., 2015; Mandal et al., 2010). Subsequently, rhizobia produce structurally conserved lipochitooligosaccharide Nod factors, which are recognized by the NOD-ACTOR RECEPTOR Complex of leguminous plants. Finally, leguminous plants undergo physiological and biochemical changes to prepare for the formation of rhizobial symbionts (Roy et al., 2020; Mario et al., 2015; Geurts et al., 2005). To date, the underlying potential promotion mechanism of soybean growth and nitrogen fixation by co-inoculation with *B. velezensis* and USDA110. Among the various rhizobia, the *NodD1* gene is the main regulatory factor for Nod factor biosynthesis and the symbiotic phenotype (del Cerro et al., 2015; Appelbaum et al., 1998; Gillette and Elkan, 1996). In contrast, *NodD*2 inhibits *NodD1* expression (Loh and Stacey, 2003; Machado and Krishnan, 2003; Fellay et al., 1998; Machado et al., 1998). A previous study observed that co-inoculation of *B. velezensis* S141 with USDA110 promoted the growth, nodulation, and nitrogen fixation of soybeans. It is believed to S141 promotes nodulation and nitrogen fixation by secreting IAA and cytokinins. In addition, it is possible that the S141 strain secretes other substances that are beneficial for nodulation (Sibponkrung et al., 2020). In this study, a metabolomic analysis was conducted on the fermentation broth of the PGPR *B. velezensis* 20507, and it was found that the metabolites of *B. velezensis* contained 29 flavonoids and four isoflavonoids, including rutin, but not IAA and cytokinin. This may be related to the genetic background differences between the two strains. However, the observed promotion of soybean seedling growth and nodulation is consistent with previous reports (Sibponkrung et al., 2020). Appropriate concentrations of rutin (5 and 10 mg/L) in the culture medium significantly increased the expression of *NodD1* and inhibited the expression of *NodD2* in USDA110 cells. Consistent with this, when co-cultured with *B. velezensis* 20507, the expression of *NodD1* was significantly upregulated, whereas *that of NodD2* was significantly downregulated (Figures 11A,B). Therefore, the effects of adding rutin (5 and 10 mg/L) to the culture medium and co-culturing with *B. velezensis* on *NodD1* and *NodD2* are similar. It is inferred that *B. velezensis* can enhance the expression of *NodD1* and inhibit the expression of *NodD2* by secreting flavonoids, such as rutin. In the BB treatment in this study, it was inferred that the flavonoids produced by *B. velezensis* were beneficial for symbiosis between the nitrogen-fixing bacteria *B. japonicum* and soybean roots, and the growth and nitrogen content levels of soybeans in the BB treatment were significantly higher than those in the Bj treatment. Although the limited root nodule biomass (<0.1 g) under 2–5 mM nitrogen precluded quantitative nitrogen analysis via K-360 detection, the observed nodulation frequency (Figure 4) provides preliminary evidence for differential symbiotic efficiency. Future studies employing large-scale field trials with enhanced sampling protocols may overcome biomass constraints of nitrogen quantification.

### 4.3 *Bacillus velezensis* promoted soybean growth by inhibiting the biosynthesis of flavonoids

Most studies suggest a negative correlation between flavonoid biosynthesis and auxin transport. Reduced flavonoid biosynthesis promotes auxin transport, whereas increased flavonoid synthesis hinders auxin transport (Santelia et al., 2008; Besseau et al., 2007; Peer et al., 2004). Some studies have suggested that flavonoid accumulation interferes with auxin distribution and turnover, inhibiting plant growth (Kuhn et al., 2016; Buer et al., 2013; Brown et al., 2001). By comparing the growth of different branches of flavonoid pathway mutants, Jeon et al. (2022) suggested that inhibition of flavonoid biosynthesis promotes plant growth, whereas promotion of flavonoid biosynthesis leads to growth inhibition. Studies have also been conducted from various perspectives. Li et al. (2010) suggested that the reduced growth associated with lignin biosynthesis in Arabidopsis was not related to flavonoids. Flavonoids are secondary metabolites with antioxidant and radical-scavenging activities. Nakabayashi et al. (2014) suggested that the accumulation of flavonoids in plants is beneficial for improving their ability to scavenge reactive oxygen species under drought stress conditions and that the accumulation of flavonoids does not inhibit plant growth. Therefore, the intrinsic relationship between plant growth and flavonoid biosynthesis remains unclear.

The phenylpropane pathway is an important metabolic pathway in plants that mainly synthesizes phenylpropane compounds such as flavonoids, soluble phenolic esters, and biopolymer precursors (Tohge et al., 2018). Flavonoids are widely distributed in plants, and are the main source of pigments that accumulate in various plant parts. Flavonoid synthesis begins with the phenylpropanoid metabolic pathway, which generates chalcones through a series of enzyme-catalyzed reactions and converts them into various flavonoid compounds. Several enzymes play crucial roles in flavonoid biosynthesis, including PAL, 4-coumaric coenzyme A ligase (4CL), chalcone synthase (CHS), and chalcone isomerase (CHI) (Wang et al., 2018; Wang et al., 2019). Compared with a single inoculation of rhizobia, co-inoculation with *Rhizobium* spp. and *Azospirillum* spp. can increase the number of root nodules by increasing the number of root hairs and the amount of flavonoids secreted by roots (Remans et al., 2008a, 2008b). Previous studies have found that when the key enzyme involved in the synthesis of flavonoids in *Medicago truncatula*, the chalcone synthase (CHS) gene, is disrupted, the inhibitory effect of rhizobia inoculation on local auxin transport in *Medicago truncatula* roots disappears and the plant cannot form nodules (Wasson et al., 2006, 2009). In paired comparisons of BB and Bj, 5,367 DEGs were identified. Among these, the number of downregulated genes was much higher than upregulated genes, with a quantity 1.32 times that of upregulated genes. The significant enrichment results of the KEGG indicated that the downregulated genes were significantly enriched in KEGG pathways, such as phenylpropanoid biosynthesis (ko00940), flavonoid biosynthesis (ko00941), and isoflavonoid biosynthesis (ko00943). Among the significantly enriched KEGG pathways, most of the key DEGs involved in flavonoid biosynthesis were downregulated (Supplementary Table S9), including *PAL*, *CHS*, *CHI*, *4CL*, *CHR2*, and *CHR6*. Therefore, it is believed that inoculation with the PGPR *B. velezensis* inhibited the biosynthesis of flavonoids in soybeans, which may be related to the feedback inhibition caused by the production of flavonoids by *B. velezensis*. Nitrogen is the most essential mineral nutrient for plant growth and development, and NO_3_^−^ is an important form of nitrogen absorption and utilization in plants. *NPF* genes are involved in the absorption, regulation, transport, and distribution of nitrate and nitrogen in plants (Sakuraba et al., 2021). Therefore, NPF genes play an important role and have practical value in improving and enhancing crop nitrogen utilization efficiency and yield-related traits. In this study, six DEGs encoding *NPF* were identified, which were upregulated 1.49–4.01-fold (Supplementary Table S9). In paired comparisons, DEGs were significantly enriched in the KEGG pathway ko04075 (Plant hormone signal transduction), in which most DEGs encoding GH3 and SAUR were upregulated. GH3 catalyzes the binding of auxin to amino acids, forming a complex that serves as an auxin storage reservoir. When the concentration of auxin is too low, the auxin-amino acid complex is hydrolyzed by proteolytic enzymes into auxin, which participates in the auxin signaling pathway and regulates the dynamic balance of auxin in plants (Bao et al., 2024). SAUR plays an important regulatory role in plant cell elongation and growth (Xu et al., 2023; Xu et al., 2017). In this study, a set of DEGs encoding *GH3 and SAUR* were upregulated by 1.13–3.02-fold (Supplementary Table S9). *B. velezensis* has been suggested to promote the growth of soybean seedlings by inhibiting the synthesis of soybean flavonoids, regulating auxin homeostasis and root elongation.

## 5 Conclusion

The enhanced growth performance of soybean seedlings in the BB treatment compared to Bj stems from three interrelated mechanisms. First, despite *B. velezensis*’ broad antimicrobial activity, it establishes a metabiotic relationship with *B. japonicum*, enhancing rhizobial proliferation through non-antagonistic interactions. Second, *B. velezensis*-secreted flavonoids (notably rutin) orchestrate nodulation in *B. japonicum* USDA110 via dual regulatory effects: activating *NodD1* while suppressing *NodD2* expression. This transcriptional reprogramming elevates *NSP2* levels, triggering NOD factor signaling that initiates symbiosis, coupled with upregulated nitrate transporter (*NPF*) genes that enhance nitrogen assimilation efficiency, thereby stimulating chlorophyll biosynthesis and photosynthetic capacity. Third, bacterial flavonoids induce feedback inhibition of root flavonoid biosynthesis through downregulated phenylpropanoid genes (*PAL*, *CHS*, *CHI*), concomitant with upregulated auxin-responsive genes (*GH3s*, *SAURs*), potentially synergizing plant growth through phytohormone modulation. While these findings from 30-day-old seedlings in vermiculite systems provide mechanistic insights, field validation with mature plants under agricultural conditions remains essential. While bacterial transcriptomics would be informative, our experimental design prioritized host plant responses, and further full transcriptomic analysis of bacterial responses is planned for our next study. Ongoing CRISPR-Cas9 engineering of *B. velezensis* flavonoid mutants will conclusively map microbial metabolic contributions. This study advances multi-strain biofertilizer design by demonstrating how tailored microbial consortia can synergistically enhance legume productivity through complementary biochemical pathways.

## Data Availability

The raw transcriptome sequencing data of soybean have been deposited in the national center for biotechnology information (NCBI) database under the accession number PRJNA1152285. The genome sequence of *B. velezensis* have been deposited in NCBI under the accession number PRJNA981422. Other relevant data supporting the findings of this study are available in this article and its associated Supplementary material.

## Footnotes

^1^ https://www.ncbi.nlm.nih.gov/SRA

## Supplementary material

Supplementary Table S1 PCR primer sequences.

Supplementary Table S2 Statistics of raw data information for each sample.

Supplementary Table S3 Statics of clean reads mapping.

Supplementary Table S4 DEGs in paired comparison of BB vs Bj.

Supplementary Table S5 GO enrichment of DEGs in BB vs Bj.

Supplementary Table S6 KEGG enrichment of DEGs in BB vs Bj.

Supplementary Table S7 Gene annotation of B. velezensis 20507.

Supplementary Table S8 Mass spectrometry identification results of fermentation broth of *B. velezensis* 20507.

Supplementary Table S9 Interested genes and their expression in paired comparison of BB vs Bj.
