# Supplementary material for: Bacillus velezensis 20507 promotes symbiosis between Bradyrhizobium japonicum USDA110 and soybean by secreting flavonoids
Source: Front Microbiol. 2025 Mar 27;16:1572568. doi: 10.3389/fmicb.2025.1572568 (PMC11983421; doi:10.3389/fmicb.2025.1572568)
Supplement: Supplementary Table S9 — Interested genes and their expression in paired comparison of BB vs Bj. [file Table_9.DOCX]

**Table S23**. Interested genes and their expression in paired comparison of BB vs Bj

| Gene ID | MeanTPM (BB) | MeanTPM  (Bj) | log_2_FoldChange | pValue | Annotation |
| --- | --- | --- | --- | --- | --- |
| Glyma.02G309300 | 2.46 | 15.02 | -2.61 | 2.29E-127 | PAL |
| Glyma.03G181600 | 3.47 | 19.49 | -2.49 | 1.55E-145 | PAL |
| Glyma.19G182300 | 19.19 | 66.56 | -1.79 | 0 | PAL |
| Glyma.03G181700 | 2.52 | 32.64 | -3.69 | 0 | PAL |
| Glyma.13G145000 | 60.55 | 177.49 | -1.55 | 0 | PAL |
| Glyma.10G058200 | 56.60 | 157.24 | -1.47 | 0 | PAL |
| Glyma.11G011500 | 56.00 | 133.33 | -1.25 | 1.63E-192 | CHS |
| Glyma.05G153200 | 7.37 | 31.39 | -2.09 | 1.67E-89 | CHS2 |
| Glyma.08G109500 | 28.67 | 128.43 | -2.16 | 0 | CHS1 |
| Glyma.08G109300 | 4.39 | 26.11 | -2.57 | 8.95E-278 | CHS3 |
| Glyma.08G110460 | 8.91 | 53.83 | -2.60 | 0 | CHS3 |
| Glyma.08G110300 | 8.53 | 54.99 | -2.69 | 3.88E-276 | CHS3 |
| Glyma.08G109200 | 10.05 | 84.71 | -3.078 | 0 | CHS5 |
| Glyma.08G110500 | 10.93 | 98.43 | -3.17 | 0 | CHS4 |
| Glyma.08G110701 | 8.81 | 80.95 | -3.19 | 0 | CHS4 |
| Glyma.08G110340 | 3.97 | 38.17 | -3.26 | 0 | CHS5 |
| Glyma.02G130400 | 1.46 | 21.04 | -3.85 | 1.72E-132 | CHS6 |
| Glyma.08G109400 | 4.93 | 72.17 | -3.87 | 0 | CHS1 |
| Glyma.09G075200 | 0.71 | 10.51 | -3.88 | 2.36E-77 | CHS6 |
| Glyma.08G110420 | 1.08 | 17.68 | -4.04 | 0 | CHS1 |
| Glyma.01G091400 | 0.95 | 23.01 | -4.60 | 3.23E-162 | CHS6 |
| Glyma.20G241500 | 123.64 | 289.33 | -1.23 | 5.63E-241 | CHI |
| Glyma.20G241700 | 0.95 | 2.75 | -1.53 | 9.28E-08 | CHI |
| Glyma.04G222400 | 26.01 | 76.33 | -1.55 | 1.41E-154 | CHI |
| Glyma.06G143000 | 102.46 | 367.12 | -1.84 | 0 | CHI |
| Glyma.13G082300 | 0.03 | 0.25 | -2.96 | 0.015599 | F3H |
| Glyma.01G232400 | 7.30 | 25.53 | -1.81 | 2.44E-154 | 4CL |
| Glyma.17G064400 | 0.13 | 0.00 | 10.34 | 1.15E-03 | 4CL |
| Glyma.11G010500 | 2.67 | 18.71 | -2.81 | 3.79E-146 | 4CL |
| Glyma.15G001700 | 5.15 | 16.73 | -1.70 | 6.59E-76 | 4CL |
| Glyma.16G219500 | 9.22 | 4.44 | 1.05 | 9.01E-19 | CHR2 |
| Glyma.02G307300 | 20.11 | 46.50 | -1.21 | 3.12E-59 | CHR6 |
| Glyma.13G081700 | 7.07 | 2.69 | 1.39 | 4.01E-16 | NSP2 |
| Glyma.06G110800 | 0.94 | 0.46 | 1.03 | 0.002707 | NSP2 |
| Glyma.17G115300 | 8.10 | 0.50 | 4.01 | 1.15E-71 | NPF6.2 |
| Glyma.13G167700 | 7.73 | 1.98 | 1.97 | 1.29E-38 | NPF6.2 |
| Glyma.14G138200 | 7.69 | 2.04 | 1.91 | 1.66E-33 | NPF1.2 |
| Glyma.11G235200 | 1.08 | 0.30 | 1.87 | 2.77E-06 | NPF5.1 |
| Glyma.03G123000 | 0.24 | 0.08 | 1.57 | 0.014025 | NPF3.1 |
| Glyma.03G122500 | 3.87 | 1.37 | 1.49 | 1.98E-12 | NPF3.1 |
| Glyma.12G141000 | 27.31 | 12.46 | 1.13 | 1.53E-62 | GH3 |
| Glyma.02G154600 | 0.24 | 0.07 | 1.80 | 0.016087 | GH3 |
| Glyma.01G190600 | 2.54 | 1.03 | 1.30 | 8.59E-07 | GH3 |
| Glyma.14G217800 | 1.94 | 0.77 | 1.34 | 1.27E-05 | SAUR |
| Glyma.01G078200 | 0.57 | 0.24 | 1.24 | 0.017182 | SAUR |
| Glyma.13G143100 | 0.50 | 0.08 | 2.72 | 0.003212 | SAUR |
| Glyma.06G007100 | 0.67 | 0.19 | 1.82 | 0.021688 | SAUR |
| Glyma.15G182300 | 0.90 | 0.15 | 2.60 | 7.58E-05 | SAUR |
| Glyma.12G035800 | 28.05 | 8.62 | 1.70 | 1.21E-42 | SAUR |
| Glyma.04G007100 | 0.52 | 0.06 | 3.02 | 0.004773 | SAUR |
| Glyma.09G222300 | 12.41 | 4.12 | 1.59 | 1.34E-14 | SAUR |
|  |  |  |  |  |  |

Note: PAL: phenylalanine ammonia-lyase; CHS: chalcone synthase; CHI: Chalcone isomerase; F3H: Flavanone 3-hydroxylase; 4CL: 4-coumarate-CoA ligase; CHR: chalcone reductase; NSP: Nodulation Signaling Pathway; NPF: NITRATE TRANSPORTER 1/PEPTIDE TRANSPORTER FAMILY.GH3: GRETCHEN HAGEN3; SAUR: Small auxin-up RNAs.
